# Impact of intramuscular adipose tissue content on short- and long-term outcomes of hepatectomy for colorectal liver metastasis: a retrospective analysis

**DOI:** 10.1186/s12957-020-01836-5

**Published:** 2020-04-07

**Authors:** Nobutoshi Horii, Yu Sawda, Takafumi Kumamoto, Nobuhiro Tsuchiya, Takashi Murakami, Yasuhiro Yabushita, Yuki Honma, Ryusei Matsuyama, Daisuke Morioka, Hirotoshi Akiyama, Itaru Endo

**Affiliations:** Department of Gastroenterological Surgery, Yokohama City University, 3-9 Fukuura, Kanazawa-Ku, Yokohama, 236-0004 Japan

**Keywords:** Colorectal cancer, Liver metastasis, Intramuscular adipose tissue content (IMAC), Sarcopenia, Prognostic factor

## Abstract

**Background:**

Numerous reports regarding sarcopenia have focused on the quantity of skeletal muscle. In contrast, the impact of the quality of skeletal muscle has not been well investigated.

**Methods:**

A retrospective analysis of 115 patients who underwent initial hepatectomy for colorectal liver metastasis between January 2009 and December 2016 in our hospital was performed. Intramuscular adipose tissue content (IMAC) was used to evaluate the quality of skeletal muscle by analysing computed tomography (CT) images at the level of the umbilicus. The impact of poor skeletal muscle quality on short-term and long-term outcomes after hepatectomy for colorectal liver metastasis was analysed.

**Results:**

Patients were divided into two groups (high IMAC and normal IMAC) according to their IMAC values, and their backgrounds were compared. There were no significant differences in most factors between the two groups. However, both body mass index (*P* = 0.030) and the incidence of postoperative complications of Clavien-Dindo grade 3 or worse (*P* = 0.008) were significantly higher in the high-IMAC group. In multivariate analyses, an operative blood loss > 600 ml (*P* = 0.006) and high IMAC (*P* = 0.008) were associated with postoperative complications of Clavien-Dindo grade 3 or worse. Overall survival and recurrence-free survival were significantly lower (*P* < 0.001 and *P* = 0.045, respectively) in the high-IMAC group than in the normal IMAC group. In multivariate analyses for poor overall survival, high IMAC was associated with poor overall survival (*P* < 0.001).

**Conclusions:**

IMAC is a prognostic factor for poor short- and long-term outcomes in patients with colorectal liver metastasis.

## Introduction

The loss of skeletal muscle (sarcopenia) has been observed in various pathological conditions and is considered to be the result of various factors [1, 2]. Sarcopenia has recently been reported to be a prognostic factor for poor short- and long-term outcomes in several cancers; these studies [3–8] assessed skeletal muscle mass using single-slice computed tomography (CT), but did not evaluate skeletal muscle quality.

To define sarcopenia, the strength and function of the skeletal muscle, in addition to muscle mass, must be evaluated. Changes in intramuscular adipose tissue (IMAT) with ageing have been reported to relate to weak and poor skeletal muscle function [7]. In addition, it has been reported that intramuscular adipose tissue content (IMAC), originally reported to be associated with the severity of non-alcoholic steatohepatitis (NASH), also reflects the quality of skeletal muscle [9, 10]. Therefore, IMAC has attracted attention as a new parameter of sarcopenia. IMAC was associated with poor prognosis after living-donor transplantation [11] and after hepatectomy for hepatocellular carcinoma [12]. However, to the best of our knowledge, the clinical significance of IMAC in patients with colorectal liver metastasis (CRLM) has not been reported [13].

Colorectal cancer (CRC), one of the most common cancers, remains the main cause of death in Japan and worldwide [14]. Moreover, approximately 25% of colorectal cancer patients are reported to have liver metastasis upon diagnosis, and there is a high rate of recurrence after hepatectomy [15, 16]. Hepatectomy is currently considered to be the most effective option for patients with liver metastases [17, 18].

Evaluating skeletal muscle area by CT imaging has been reported to be an inadequate method for assessing sarcopenia [12]. As CT imaging cannot distinguish muscle from adipose tissue, tissues with low muscle mass and high adipose tissue content could be deemed to have normal skeletal muscle area when measured by CT. In contrast, because IMAC reflects the quality of skeletal muscle, it has been suggested to be a better prognostic factor for sarcopenia in several diseases [12, 13]. However, there have been few studies on the utility of IMAC as a prognostic factor, and a significant association between CRLM and IMAC has not been reported.

In the present retrospective study, we evaluated skeletal muscle quality as reflected by IMAC as a new parameter of sarcopenia, with the aim of clarifying the relationship between IMAC and postoperative short- and long-term outcomes after hepatectomy for CRLM.

## Methods

### Patients and methods

The study used data for 189 patients who underwent initial hepatectomy for CRLM, from January 2009 to December 2016, at the Yokohama City University Hospital. The following were excluded: 44 cases which required severe surgical invasion related to two-staged hepatectomy, 26 cases which lacked a CT image at the level of the umbilicus before surgery, and four cases for which a liver-first approach was performed. After exclusions, the medical records of the remaining 115 patients with CRLM were reviewed. This retrospective study was approved by the ethics committee of Yokohama City University (B190300006).

Hepatectomy was performed using a Cavitron Ultrasonic Surgical Aspirator (CUSA) and a bipolar cautery device equipped with a channel for water dripping. An intermittent Pringle manoeuvre and selective vascular clamping were utilized as necessary. Postoperative pathological diagnoses were performed to measure tumour size, tumour number, microvascular invasion, and tumour differentiation. All patients were followed up every 3 months after surgery, and tumour markers and CT scans were evaluated.

### Imaging analysis

All CT imaging before surgery was performed with a multi-detector computed tomography scanner (Aquilion CXL and Aquilion PRIME, Canon Medical Systems, Otawara, Japan; SOMATOM Definition Flash, Siemens Healthcare, Forchheim, Germany). IMAC was calculated as follows: IMAC = region of interest (ROI) of the multifidus muscle (Hounsfield units)/ROI of subcutaneous fat (Hounsfield units) [12, 13].

**Fig. 1:**
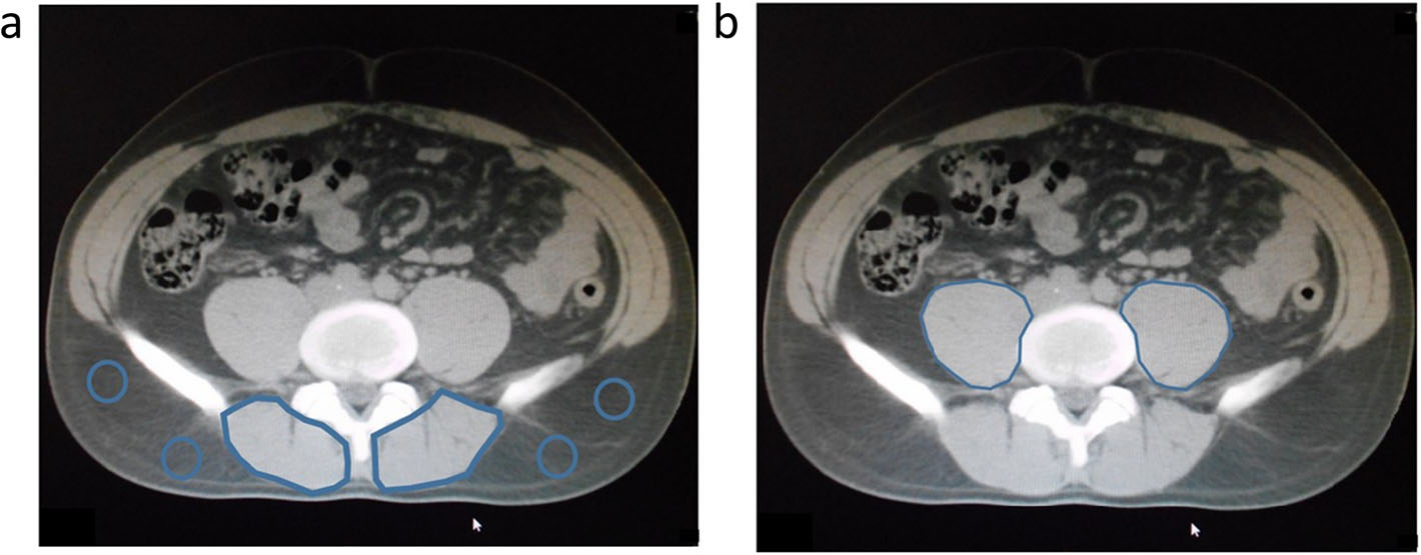
**a** Cross-sectional computed tomography imaging of subcutaneous fat area and subfascial muscular tissue in multifidus muscle. **b** Bilateral psoas muscles at level of umbilicus

On the preoperative plain CT, the subfascial muscular tissues in the multifidus muscle were traced at the level of the umbilicus, and the CT values (in Hounsfield units) were calculated using the server in our hospital (Fig. 1a). CT values of the subcutaneous fat tissue were calculated using four circles traced on the subcutaneous fat area away from major vessels (Fig. 1a) at the level of the umbilicus. The average value of these four ROIs was defined as the ROI of the subcutaneous fat.

In general, IMAC tended to be a negative value. When the skeletal muscle contained more fat tissue, IMAC tended to be higher. Therefore, a higher IMAC value represents poorer skeletal muscle quality.

Skeletal muscle mass was evaluated using the psoas muscle index (PMI) at the level of the umbilicus. The bilateral psoas muscle area was evaluated using manual tracing (Fig. 1b). The PMI was calculated by normalizing these cross-sectional areas to height (cm^2^/m^2^) [12, 13].

In this study, due to the differences in the IMAC values of male and female patients, the cut-off point for each sex was set using a receiver operating characteristic curve (ROC curve).

The dependent variable was postoperative overall survival. The cut-off value was − 0.335 for men and − 0.258 for women. Patients with a value higher than or equal to the cut-off value were classified in high-IMAC group (H group), and the remainder were classified in the normal-IMAC group (N group).

### Analysed parameters

Overall survival rate (OS) and recurrence-free survival rate (RFS) after hepatectomy for CRLM were evaluated for the patients classified according to IMAC. In addition, the following variables were analysed as prognostic factors: patient age, sex, body mass index (BMI), serum albumin, Onodera’s prognostic nutritional index (PNI) [19], modified Glasgow prognostic score (mGPS) [20], American Society of Anesthesiologists Classification score (ASA score), PMI, carcinoembryonic antigen (CEA) level, serum carbohydrate antigen 19-9 (CA19-9) level, tumour size, number of tumours, tumour location, primary tumour location, surgical procedure, detection of metastasis at hepatectomy, operation time, operation bleeding, surgical margin as diagnosed by pathologists, perioperative chemotherapy, and the occurrence of postoperative complications. Postoperative complications were assessed using the Clavien-Dindo classification system [21, 22].

### Statistical analyses

Values are presented as median (range) or number (percentage). Differences were assessed using Mann-Whitney *U* tests for numerical variables and Fischer’s exact probability tests for categorical variables. Survival was assessed using Kaplan-Meier life tables, and differences in survival were evaluated using Gehan-Breslow-Wilcoxon tests. A two-tailed *P* value (*P*) of < 0.05 was considered significant. Statistical analyses were performed using SPSS commercial statistics software version 22 (IBM, Armonk, NY, USA).

## Results

### Patient characteristics

Baseline characteristics of the 115 patients in this study are summarized in Table 1. The median patient age was 67 years. Almost all patients had an ASA score of 1 or 2. Twenty patients presented with metastasis to other organs at hepatectomy.

**Table 1:** Patient characteristics in this study

	Total (*n* = 115)
Age at surgery (years)	
Median (IQR)	67 (27–85)
Gender, *n* (%)	
Male	79 (69)
Female	36 (31)
Serum albumin level (g/dl)	
Median (IQR)	4.1 (3.2–5.0)
ASA score, *n* (%)	
1	32 (28)
2	75 (65)
3	8 (7)
PNI	
Median (IQR)	47.7 (35.9–59.2)
CRP (mg/dl)	
Median (IQR)	0.90 (0.01–4.24)
mGPS, *n* (%)	0
0	93 (81)
1	18 (16)
2	4 (3)
BMI	
Median (IQR)	21.3 (14.7–30.4)
Primary tumour location, *n* (%)	
Colon	77 (67)
Rectum	38 (33)
Tumour location, *n* (%)	
Unilobe	16 (14)
Bilobe	99 (86)
Tumour size (mm)	
Median (IQR)	28 (5–120)
Number of tumours	
Median (IQR)	3 (1–30)
Detection of metastasis, *n* (%)	
Synchronous	60 (52)
Metachronous	
CEA (ng/dl)	
Median (IQR)	5.5 (1.1–855)
CA19-9 (mU/l)	
Median (IQR)	26 (1–2368)
Preoperative chemotherapy, *n* (%)	
Yes	71 (62)
No	44 (38)
Postoperative chemotherapy, *n* (%)	
Yes	48 (42)
No	67 (58)
Other organ metastasis at hepatectomy, *n* (%)	
Yes	20 (17)
Surgical margin, *n* (%)	
Positive	21 (18)
Negative	94 (82)
Surgical procedure, *n* (%)	
Major resection	34 (30)
Minor resection	81 (70)
Synchronous resection for primary tumour, *n* (%)	
Yes	9 (8)
Operation time (min)	
Median (IQR)	431 (198–797)
Operative blood loss (ml)	
Median (IQR)	524 (0–2257)
Postoperative complications, *n* (%)	
Clavien-Dindo grade II	20 (17)
Clavien-Dindo grade III or IV	12 (10)

*ASA score* American Society of Anesthesiologists classification score, *BMI* body mass index, *CA19-9* carbohydrate antigen 19-9, *CEA* carcinoembryonic antigen, *CRP* C-reactive protein, *IQR* interquartile range, *mGPS* modified Glasgow prognostic score, *PNI* prognostic nutritional index

Comparisons between the IMAC H and N groups are shown in Table 2. BMI was significantly lower in the N group than in the H group (*P* = 0.030), but there were no significant differences in other tumour- and patient-related factors. Moreover, postoperative complications (Clavien-Dindo grade 3 or worse) were significantly more frequent in the H group than in the N group (17.1% vs. 1.9%; *P* = 0.011). PMI, an indicator of skeletal muscle mass, was not significantly different between the two groups.

**Table 2:** Comparison of patient backgrounds between normal and high IMAC groups

	H group (*n* = 64)	N group (*n* = 51)	*P* value
Age (years)			
Median (IQR)	68 (40–85)	65 (27–80)	0.381
Gender, *n* (%)			
Male	47 (72)	32 (63)	0.232
Female	17 (28)	19 (27)	
Serum albumin level (g/dl)			
Median (IQR)	4.1 (3.3–5.0)	4.1 (3.2–4.9)	0.378
ASA score, *n* (%)			
1	20 (31)	12 (24)	0.646
2	40 (63)	35 (68)	
3	4 (6)	4 (8)	
PNI			
Median (IQR)	47.2 (38.7–58.3)	49.4 (35.9–59.2)	0.113
CRP			
Median (IQR)	0.1 (0.01–7.24)	0.08 (0.01–4.14)	0.504
mGPS, *n* (%)			
0	54 (84)	39 (76)	0.373
1	9 (14)	9 (18)	
2	1 (2)	3 (6)	
BMI			
Median (IQR)	21.9 (14.7-30.4)	20.7 (16.1-27.4)	0.030
Primary tumour location, *n* (%)			
Colon	44 (69)	33 (65)	0.693
Rectum	20 (31)	18 (35)	
Tumour location, *n* (%)			
Unilobe	30 (47)	19 (27)	0.345
Bilobe	34 (53)	32 (63)	
Tumour size (mm)			
Median (IQR)	33.5 (5–120)	27 (7–80)	0.156
Number of tumours			
Median (IQR)	3 (1–30)	4 (1–12)	0.677
Detection of metastasis, *n* (%)			
Synchronous	30 (47)	30 (59)	0.260
Metachronous	34(53)	21 (41)	
CEA (ng/dl)			
Median (IQR)	8.3 (1.3–855)	4.3 (1.1–718)	0.504
CA19-9 (mU/l)			
Median (IQR)	27 (1–2368)	14 (1–984)	0.477
Preoperative chemotherapy, *n* (%)			
Yes	41 (64)	30 (59)	0.700
No	23 (36)	21 (41)	
Postoperative chemotherapy, *n* (%)			
Yes	24 (38)	24 (47)	0.302
No	40 (62)	27 (53)	
Other organ metastasis, *n* (%)			
Yes	11 (17)	9 (17)	1.000
Surgical margin, *n* (%)			
Yes	12 (18)	9 (18)	1.000
No	52 (82)	42 (82)	
Surgical procedure, *n* (%)			
Major resection	20 (39)	14 (28)	0.686
Minor resection	44 (61)	37 (72)	
Synchronous resection for primary tumour			
Yes	4 (8)	5 (8)	0.481
Operation time (min)			
Median (IQR)	427 (198–783)	432 (229–797)	0.934
Operative blood loss (ml)			
Median (IQR)	515 (0–2257)	526 (10–1975)	0.934
PMI			
Median (IQR)			
Male	6.2 (1.8–10.1)	6.1 (3.8–10.1)	0.892
Female	4.4 (2.2–10.7)	4.4 (2.3–10.7)	1.000
Post-op complication ≥ C-D grade 2			
Yes, *n* (%)	20 (31)	12 (23)	0.407
Post-op complication ≥ C-D grade 3			
Yes, *n* (%)	11 (17)	1 (1.9)	0.011

*ASA score* American Society of Anesthesiologists classification score, *BMI* body mass index, *CA19-9* carbohydrate antigen 19-9, *CEA* carcinoembryonic antigen, *CRP* C-reactive protein, *IQR* interquartile range, *mGPS* modified Glasgow prognostic score, *PMI* psoas muscle index, *PNI* prognostic nutritional index, *Post-op* postoperative

### Impacts of IMAC on postoperative complications

As IMAC is considered to be associated with short-term prognosis, other risk factors for postoperative complications (Clavien-Dindo grade 3 or worse) were analysed. Univariate analysis and multivariate logistic regression analysis with backward elimination for postoperative complications of Clavien-Dindo grade 3 or worse are shown in Table 3.

**Table 3:** Odds ratios from univariate and multivariate analysis of postoperative complications of Clavien-Dindo grade 3 or worse

	Univariate			Multivariate		
	Odds ratio	95% CI	*P* value	Odds ratio	95% CI	*P* value
Age ≥ 70 years	1.125	0.334–3.787	1.000			
Male	1.414	0.359–5.669	0.849			
ASA score 2 or 3	2.055	0.425–9.942	0.362			
PNI < 45	1.040	0.261–4.143	0.956			
mGPS 1 or 2	2.361	0.641–8.694	0.186			
BMI < 22 kg/m^2^	0.472	0.140–1.590	0.218			
Bilobed tumour	1.552	0.439–5.480	0.492			
Tumour size ≥ 30 mm	3.717	0.951–14.53	0.046	2.633	0.425–7.359	0.203
Number of tumours ≥ 4	0.327	0.084–1.277	0.094			
Metachronous occurrence	0.757	0.226–2.541	0.652			
CEA ≥ 10 ng/dl	1.512	0.456–5.013	0.497			
CA19-9 ≥ 20 mU/l	1.887	0.535–6.658	0.318			
Major resection	2.697	0.797–9.000	0.101			
Synchronous resection for primary tumour	1.008	0.123–9.460	0.945			
Operation time ≥ 480 min	3.407	1.002–11.583	0.040	1.768	0.592–11.703	0.433
Operative blood loss > 600 ml	8.205	1.708–39.420	0.003	9.564	1.913–47.811	0.006
H group	10.377	1.292–83.337	0.008	12.326	1.478–102.784	0.020
Low PMI group	0.504	0.129–1.974	0.318			

*ASA score* American Society of Anesthesiologists classification score, *BMI* body mass index, *CA19–9* carbohydrate antigen 19-9, *CEA* carcinoembryonic antigen, *CI* confidence interval, *H group* high intramuscular adipose tissue content group, *mGPS* modified Glasgow prognostic score, *PMI* psoas muscle index, *PNI* prognostic nutritional index

In the univariate analysis, a tumour size > 30 mm (*P* = 0.046), operation time > 480 min (*P* = 0.040), operative blood loss > 600 ml (*P* = 0.003), and the H group (*P* = 0.020) were significantly associated with a postoperative complication of Clavien-Dindo grade 3 or worse. The PMI cut-off values were set using an ROC curve, and patients with low PMI were classified as the low PMI group (PMI cut-offs: men, 6.0; women, 4.0). No significant association was found between postoperative complications and the low PMI group.

### RFS after hepatectomy

**Fig. 2:**
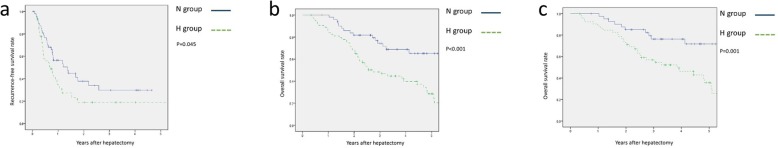
Comparison of survival of patients with high and normal intramuscular adipose tissue content. **a** Recurrence-free survival. **b** Overall survival. **c** Overall survival, excluding cases with metastasis from other organs at the time of hepatectomy. H group: patients with high intramuscular adipose tissue content. N group: patients with normal intramuscular adipose tissue content

RFS after hepatectomy for CRLM was significantly lower in the IMAC H group than in the N group (*P* = 0.045; Fig. 2a). Twenty patients had metastases to other organs and were excluded from this comparison.

### OS after hepatectomy for CRLM

OS was significantly lower in the IMAC H group than in the N group (*P* < 0.001; Fig. 2b). Moreover, when cases with metastasis from other organs at the time of hepatectomy were excluded, OS was significantly worse in the H group (*P* = 0.001; Fig. 2c).

We analysed several other prognostic factors for OS. The results of univariate analyses and multivariate analysis with backward elimination are summarized in Table 4 In univariate analyses, bilobed tumour (*P* = 0.010), tumour size > 30 mm (*P* = 0.009), number of tumours > 3 (*P* = 0.026), other-organ metastasis at hepatectomy (*P* < 0.001), CEA > 10 ng/dl (*P* = 0.001), postoperative complications of Clavien-Dindo grade 3 or worse (*P* < 0.001), and the H group (*P* < 0.001) were significantly associated with poor OS. In the multivariate analysis, bilobed tumour (*P* = 0.002), other-organ metastasis (*P* = 0.001), CEA > 10 ng/dl (*P* = 0.025), and the H group (*P* < 0.001) were associated with poor OS. As a high IMAC was associated with postoperative complications of Clavien-Dindo grade 3 or worse, postoperative complications were excluded in the multivariate analysis as confounding factors.

**Table 4:** Univariate and multivariate analyses for overall survival

	Univariate analysis			Multivariate analysis		
	Odds ratio	95% CI	*P* value	Odds ratio	95% CI	*P* value
Age ≥ 70 years	0.864	0.506–1.478	0.594			
Male	1.328	0.746–2.363	0.334			
ASA score 2 or 3	1.497	0.825–2.717	0.181			
PNI < 45	1.167	0.639–2.130	0.616			
mGPS 1 or 2	1.304	0.702–2.420	0.399			
BMI < 22 kg/m^2^	0.612	0.364–1.030	0.062			
Bilobe tumour	2.039	1.176–3.535	0.010	2.367	1.357–4.128	0.002
Primary tumour rectum	0.798	0.452–1.406	0.433			
Tumour size ≥ 30 mm	1.989	1.177–3.361	0.009			
Number of tumours ≥ 4	1.789	1.063–3.013	0.026			
Metachronous	1.003	0.597–1.684	0.991			
Other organ metastasis at Hx	2.714	1.518–4.855	< 0.001	2.891	1.557–5.371	0.001
Preoperative chemotherapy	1.231	0.716–2.118	0.452			
Postoperative chemotherapy	0.698	0.408–1.194	0.698			
CEA ≥ 10 ng/dl	2.356	1.402–3.958	0.001	1.838	1.078–3.133	0.025
CA19-9 ≥ 20 mU/l	1.578	0.928–2.684	0.089			
Major resection	1.306	0.746–2.286	0.348			
Synchronous resection for primary tumour	0.981	0.354–2.719	0.970			
Operation time ≥ 480 min	1.174	10.683–2.018	0.562			
Operative blood loss > 600 ml	1.350	0.683–2.018	0.254			
Po complication ≥ C-D grade 3	3.568	1.819–7.000				
H group	2.842	1.577–5.123	< 0.001	15.280	1.478–102.784	< 0.001
Low PMI group	0.850	0.497–1.453	0.552			

*ASA score* American Society of Anesthesiologists classification score, *BMI* body mass index, *CA19-9* carbohydrate antigen 19-9, *CEA* carcinoembryonic antigen, *CI* confidence interval, *H group* high intramuscular adipose content group, *Hx* hepatectomy, *mGPS* modified Glasgow prognostic score, *PMI* psoas muscle index, *PNI* prognostic nutritional index, *Po complication ≥ C-D grade 3* postoperative complication as Clavien-Dindo grade 3 or more severe

## Discussion

Skeletal muscle loss (sarcopenia) has been identified as a prognostic factor for several malignant diseases [3–8]. However, many studies have focused only on skeletal muscle mass, as assessed by CT of skeletal muscle area. In contrast, insufficient attention has been given to the deterioration of muscle quality that is associated with muscle fat deposition [3–8].

The usefulness of IMAC in hepatectomy for hepatocellular carcinoma (HCC) has been reported [12]. Our study was therefore conducted to assess the possible usefulness of IMAC in CRLM patients undergoing hepatectomy. In HCC, chronic hepatitis often exists in the background liver, and progression of fibrosis has been observed in the background liver. On the other hand, patients with CRLM rarely have chronic hepatitis in the background liver, and HCC and CRLM are considered to be different. Therefore, we believe that our finding—that IMAC is useful for predicting the prognosis of CRLM—is novel. To the best of our knowledge, our study is the first to find a significant association between IMAC and short- and long-term prognosis in patients with CRLM.

The utility of IMAC for assessing CRLM outcomes has been investigated previously [13]; in contrast to our findings, that study found that IMAC did not have a statistically significant impact on short- and long-term outcomes. The patient background data in our study are comparable with the data used in the previous study [13], and many factors show no remarkable differences between that study and ours. However, our study may have included several patients who underwent more severe surgical invasion or were more advanced cases. These differences may have contributed to the statistical significance observed in our study.

In the present study, there were no significant differences in many factors when comparing patient backgrounds between the H and N groups (Table 2). In particular, there was no significant difference between the two groups with respect to the presence or absence of metastasis to other organs at hepatectomy or with respect to tumour markers, which are considered to be strongly correlated with long-term prognosis. Furthermore, BMI, which was reported to be a poor prognostic factor by several investigative groups [23, 24], tended to be lower in the N group, which was defined as not having sarcopenia, than in the H group. Therefore, BMI was not considered useful in predicting the long-term prognosis of patients in this study. In the studies examining the clinical significance of IMAC in patients with several diseases [9, 10, 12, 13], BMI was reported to be higher in sarcopenia groups as defined by IMAC. BMI is widely used as an indicator of nutritional status because it is convenient to measure in daily clinical practice. However, evaluating BMI might not be useful for assessing nutritional status in patients with CRLM. Recently, the concept of sarcopenic obesity has been proposed. It has been suggested that sarcopenic obesity is a poor prognostic factor in several malignant diseases [25, 26]. In the present study, BMI was higher in the H group than in the N group; thus, the proportion of body fat might also be higher in the H group, suggesting that a high IMAC may be an index of sarcopenic obesity. Therefore, IMAC might be useful in assessing sarcopenic obesity.

PMI, which we used as a quantitative index of skeletal muscle, was not associated with short- or long-term outcomes. Therefore, we do not consider PMI to be an adequate index to measure the sarcopenic condition of patients. In previous studies about liver disease [9–11], PMI was reported to be insufficient as a prognostic predictor; therefore, we thought that this tendency might be reflected in our findings. To assess this possibility, we therefore focused on muscle quality, in terms of IMAC. When PMI is derived from the area of skeletal muscle, it might reflect both fat and muscle content. Thus, it is possible that actual skeletal muscle mass cannot be obtained by measuring the area of skeletal muscle. In contrast, IMAC is calculated as the ratio of skeletal muscle to subcutaneous adipose tissue, using CT values. Therefore, IMAC may reflect the sarcopenic state of a patient more accurately than measurements of skeletal muscle area. The negative impacts of skeletal muscle fat deposition include muscle weakness and associated restricted movement [27, 28]; this pathology may contribute to the high incidence of postoperative complications. Moreover, postoperative complications have been reported as long-term prognostic factors in several malignancies [29, 30]. In our study, the incidence of postoperative complications was higher in the sarcopenic H group than in the N group. This may have contributed to the poor long-term prognosis in the H group.

Although sarcopenia has been reported previously as a prognostic factor in various malignancies [3–8], its mechanism has not been fully elucidated. Skeletal muscle is maintained by the balance between protein degradation and synthesis [31]. However, in sarcopenic patients, muscle catabolism is increased [32, 33], presumably due to chronic inflammation [34–37]. The cause of chronic inflammation is attributed to the presence of cancer cells [38, 39], obesity [40–45], adipocytokines secreted from adipocytes [40–45], and the suppression of cancer immunity [39]. In the present study, BMI was higher in the H group than in the N group; therefore, the proportion of body fat might also be considered higher in the H group. Accordingly, it is possible that skeletal muscle catabolism had progressed in the sarcopenic H group. Moreover, in the H group, antitumour immunity may have been reduced for the reasons described above [39], thereby affecting the long-term prognosis of the patients. It is possible that cancer, and chronic inflammation caused by obesity, might cause fat deposition in skeletal muscle.

There was no significant difference between the two groups regarding the site of primary metastasis. A systemic decline in cancer immune function might have contributed to the increased recurrence rate. However, chronic inflammation is only one of several mechanisms related to the cause of sarcopenia. Further studies are needed to elucidate the pathological mechanisms of sarcopenia.

To improve sarcopenic conditions, nutritional interventions, such as amino acid supplementation, have been reported to be effective [27, 46]. In the future, prospective studies are needed to establish the specific nutritional interventions which may improve the postoperative prognosis of patients with CRLM and high IMAC. Moreover, as we did not measure the serum adipocytokine level, or the visceral and subcutaneous fat area, we intend to conduct further research focusing on adipocytokines and fat volumes.

The present study had several limitations. One limitation relates to the number of patients included. Although there have been some reports on the association between IMAC and malignancy [11–13], our study used fewer patients than those studies. We excluded 26 patients without umbilicus-level CT data. Further, because of differences in surgical invasiveness and treatment policies, we also excluded patients who underwent two-stage hepatectomy or prior hepatectomy. However, we plan to continue collecting patient data. A second limitation of our study is whether the IMAC and PMI cut-off values that we used were appropriate. At present, there is no consensus on the appropriate IMAC and PMI values of healthy individuals. In our study, sarcopenia was defined using ROC curve-derived cut-off values. The ROC curve is considered a reasonable way to determine cut-off values [9]. However, we hope that IMAC and PMI will be used to define sarcopenia more commonly in the future.

A third limitation is the extended observation period. Excluding four cases for which follow-up was not possible, 13 of the patients had their surgery less than 5 years ago. For these few patients, postoperative observation will continue in order gain additional insight regarding the usefulness of IMAC as a prognostic factor. A fourth limitation is the heterogeneity of the patient background factors. We included cases of metastasis to various other organs at the time of hepatectomy, and there was no standardized adjuvant chemotherapy and treatment. Twenty patients with metastasis to other organs were included in this study. However, it has been reported that the prognosis after hepatectomy for patients with resectable or controllable extrahepatic metastasis is not inferior to that of patients with liver metastasis alone [47, 48]. Moreover, because there was no significant difference in the proportion of patients with other-organ metastases between the high-IMAC group and the normal-IMAC group, we included cases with metastasis to other organs.

It has been reported that adjuvant chemotherapy after hepatectomy for CRLM does not improve prognosis [49]. We did not establish clear administration criteria for adjuvant chemotherapy in our study. Moreover, in this study, we did not actively conduct neoadjuvant chemotherapy. The feasibility of using neoadjuvant chemotherapy for CRLM is still controversial [50–52]. Although neoadjuvant chemotherapy for resectable CRLM is recommended in the National Comprehensive Cancer Network (NCCN) guidelines [53], hepatectomy without neoadjuvant chemotherapy is recommended in the Japanese guidelines [54, 55]. Therefore, we did not actively conduct neoadjuvant chemotherapy for resectable CRLM. In principle, hepatectomy was performed for patients with fewer than four metastases, without using neoadjuvant chemotherapy. Patients with four or more metastases in both lobes, major vessel invasion, or extrahepatic metastases were treated with neoadjuvant chemotherapy before hepatectomy. We hope that the feasibility of IMAC will be demonstrated in the group of patients who underwent neoadjuvant chemotherapy for resectable CRLM. We found no significant difference in the proportion of patients who received perioperative chemotherapy in groups H and N; thus, perioperative adjuvant chemotherapy was not considered to be a prognostic factor in the present study.

## Conclusions

In conclusion, high IMAC impacted the postoperative short- and long-term prognosis of patients with CRLM. Preoperative IMAC might be considered as a new selection criterion for hepatectomy in CRLM patients.

## Data Availability

The datasets used and analysed in this study are not publicly available (to maintain privacy) but are available from the corresponding author on reasonable request.

## Abbreviations

ASA score: American Society of Anesthesiologists classification score
BMI: Body mass index
CA19-9: Carbohydrate antigen 19-9
CEA: Carcinoembryonic antigen
CRC: Colorectal cancer
CRLM: Colorectal liver metastasis
CT: Computed tomography
CUSA: Cavitron Ultrasonic Surgical Aspirator
IMAC: Intramuscular adipose tissue content
IMAT: Intramuscular adipose tissue
mGPS: Modified Glasgow prognostic score
NASH: Non-alcoholic steatohepatitis
OS: Overall survival
PMI: Psoas muscle index
PNI: Prognostic nutritional index
RFS: Recurrence-free survival
ROC curve: Receiver operating characteristic curve
ROI: Region of interest
